# Risk assessment for postoperative complications in patients undergoing cardiac surgical procedures

**DOI:** 10.1590/0034-7167-2023-0127

**Published:** 2024-09-20

**Authors:** Carolina Larrosa de Almeida, Jones Sidnei Barbosa de Oliveira, Cláudia Geovana da Silva Pires, Cláudia Silva Marinho

**Affiliations:** I Universidade Federal da Bahia. Salvador, Bahia, Brazil

**Keywords:** Cardiovascular Diseases, Cardiac Surgical Procedures, Postoperative Complications, Risk Assessment, Cardiovascular Nursing., Enfermedades Cardiovasculares, Procedimientos Quirúrgicos Cardíacos, Complicaciones Posoperatorias, Medición de Riesgo, Enfermería Cardiovascular

## Abstract

**Objectives:**

to evaluate the risk of postoperative complications in cardiac patients.

**Methods:**

an evaluative study using the Tuman Score on medical records of 70 adult patients who underwent cardiac surgery at a University Hospital. The R for Windows software was used for the analyses. Descriptive statistics and bivariate analysis were employed to verify the association between the risk score and complications. The relative risk between the Tuman Score and postoperative complications was obtained through Quasi-Poisson regression, with a 95% confidence interval.

**Results:**

the majority of the patients were male (58.57%), aged between 41-64 years (50%), who underwent myocardial revascularization (50%). These patients were associated with a lower risk of postoperative complications (p=0.003), (p=0.008), and (p=0.000), respectively. High-risk patients had pulmonary complications (RR=1.32, p=0.002) and neurological complications (RR=1.20, p=0.047).

**Conclusions:**

preoperative risk assessment promotes qualified care to reduce postoperative complications.

## RESUMEN

**Objetivos:**

evaluar el riesgo de complicaciones posoperatorias en pacientes cardíacos.

**Métodos:**

investigación evaluativa, utilizando el Escore de Tuman, en historiales médicos de 70 pacientes adultos sometidos a cirugía cardíaca en un Hospital Universitario. Se utilizó el software R for Windows para los análisis. Se emplearon estadísticas descriptivas y análisis bivariados para verificar la asociación entre el escore de riesgo y las complicaciones. El riesgo relativo entre el Escore de Tuman y las complicaciones posoperatorias se obtuvo mediante regresión Quasi-Poisson, con un intervalo de confianza del 95%.

**Resultados:**

predominaron los pacientes del sexo masculino (58.57%), con edades entre 41 y 64 años (50%), que se sometieron a Revascularización del Miocardio (50%), y estos estuvieron asociados con un menor riesgo de complicaciones posoperatorias (p=0,003), (p=0,008) y (p=0,000), respectivamente. Los pacientes de alto riesgo tuvieron complicaciones pulmonares (RR=1,32 y p=0,002) y complicaciones neurológicas (RR=1,20 y p=0,047).

**Conclusiones:**

la evaluación de los riesgos preoperatorios promueve una atención de calidad para reducir las complicaciones posoperatorias.

## INTRODUCTION

In the preoperative phase of cardiac surgical procedures, it is important to estimate the probability of adverse outcomes. In Brazil, approximately 24,000 cardiac surgeries were performed in 2022. In the same year, 642 patients were hospitalized for the treatment of complications following these procedures. Compared to the previous year, there is an upward trend in the number of such surgeries, as well as an increase in postoperative complications, with 19,632 cardiac surgical procedures performed and 329 complications^(1)^. The most common complications were reduced urinary output, cardiac arrhythmias, and hypertension^(2)^.

In this context, the use of risk scores equips healthcare professionals to estimate the prognosis for patient complications. Complications arising from healthcare services have a significant impact on individual patient health, as well as on the economic burden of the healthcare system. Therefore, it is essential to implement strategies to identify patients at higher risk of complications and adopt measures to prevent or minimize them. Published studies describe several risk scores and indices used worldwide^(3-5)^. This study utilized the Tuman Score^(6)^ as it is widely used and validated by Brazilian researchers to assess risk and identify factors for postoperative complications in patients undergoing cardiac surgical procedures, with a practical method comprising easily obtainable variables.

This investigative study is important as it allows for the identification of the clinical profile of patients undergoing cardiac surgical procedures, the complications that occur postoperatively, and the estimation of the risk for these complications. This empowers the nursing team and other healthcare professionals with knowledge to develop actions aimed at improving their practices, care protocols, and individualized care plans for these patients.

Additionally, a gap in the literature was identified, where the majority of studies repeatedly identify and describe postoperative risks as adverse situations, but few provide evidence of early interventions for the prevention and control of postoperative risks.

In this context, this study is innovative and original as it applies, discusses, and disseminates a care technology, a systematic tool for early risk assessment for postoperative complications in cardiac patients, validated in Brazil and simplified for use by nursing and other healthcare professionals.

## OBJECTIVES

To evaluate the risk of postoperative complications in patients undergoing cardiac surgical procedures using the Tuman Score.

## METHODS

### Ethical Aspects

This research adhered to the guidelines of Resolutions 466/2012 and 510/2016 of the Brazilian National Health Council concerning research involving human subjects^(7-8)^ and the orientations of Circular Letter No. 039/2011/CONEP/CNS/GB/MS, which addresses the use of medical records data for research purposes in Brazil^(9)^. Patient consent was waived as this was a retrospective, document-based study. Approval was obtained from the Research Ethics Committee (CEP) of the Professor Edgar Santos University Hospital (HUPES).

### Study Design, Period, and Location

This evaluative, observational, and retrospective study investigated all patients who underwent cardiac surgical procedures at a university hospital in Salvador, Bahia, from January 1, 2019, to December 31, 2020. The university hospital, a large facility, is a reference center in the state for the treatment of cardiac patients and includes specialized services such as outpatient clinics, a cardiology ward, a cardiology intensive care unit, hemodynamics, and a surgical center. Data collection was conducted from the patients’ electronic and physical medical records between February 1 and March 31, 2021, by one of the authors, a resident nurse working in the Cardiac Surgery Clinic. This manuscript is a product of her final coursework project. The manuscript was prepared following the recommendations of the Strengthening the Reporting of Observational Studies in Epidemiology (STROBE).

### Sample and Eligibility Criteria

During the study period, 130 cardiac surgical procedures were performed, of which 70 were selected for the study. The inclusion criteria were surgeries on patients aged 18 years or older, of both sexes, who underwent: valvular surgery (replacement or repair); coronary artery bypass surgery (with or without the use of extracorporeal circulation); and combined surgery (coronary artery bypass surgery + valvular surgery). Data from patients who did not meet these eligibility criteria were excluded from the study.

### Study Protocol

Data collection was performed using patient medical records. The data of interest included hospitalization characteristics: length of stay (preoperative and postoperative duration in the Intensive Care Unit - ICU - and in the ward); and the variables (social and clinical) for calculating the Tuman Risk Score: urgency of surgery; age; sex; pre-existing health conditions (renal dysfunction; acute myocardial infarction within 3-6 months, less than 3 months, or no prior infarction; cardiac surgery; pulmonary hypertension; history of cerebrovascular disease; congestive heart failure; left ventricular dysfunction); type of cardiac surgical procedure to be performed: multiple valve replacement or myocardial revascularization associated with valve replacement; and the specific cardiac valve to be repaired: aortic or mitral^(6)^.

The Tuman Score is a risk prediction model for postoperative complications in cardiac surgical procedures, developed in the Department of Anesthesiology, Cardiology, and Thoracic Surgery in Chicago, United States, in 1992. The goal was to create a model to stratify the risk of morbidity following cardiac surgical procedures in adults using objective and readily available clinical data^(6)^.

Accordingly, the definitions of the categories to be evaluated were considered: acute myocardial infarction, pulmonary hypertension, cerebrovascular disease, congestive heart failure, left ventricular dysfunction, and renal dysfunction, as well as cardiac, pulmonary, renal, neurological, and infectious complications^(6)^.

- Acute Myocardial Infarction: Patient presenting with two or more of the following findings: elevated creatine phosphokinase-MB fraction (CKMB); new Q waves on electrocardiogram; reduced uptake of technetium pyrophosphate on myocardial scintigraphy.
- Pulmonary Hypertension: Systolic pulmonary artery pressure ≥30 mmHg as evidenced by echocardiogram or during hemodynamic study.
- Cerebrovascular Disease: History of stroke and/or vascular abnormalities diagnosed during carotid angiography.
- Congestive Heart Failure: Radiological findings consistent with pulmonary congestion or the presence of a third heart sound.
- Left Ventricular Dysfunction: Ejection fraction less than 35% on echocardiogram.
- Renal Dysfunction: Serum creatinine greater than 1.4 mg/dL (milligrams per deciliter).
- Cardiac Complications:
- Perioperative Acute Myocardial Infarction: Patient presenting with two or more of the following: elevated CKMB; new Q waves on electrocardiogram; reduced uptake of technetium pyrophosphate on myocardial scintigraphy.
- Low Cardiac Output Syndrome: Cardiac index less than two liters per minute per square meter, with the need for inotropic drugs for more than two hours and/or use of an intra-aortic balloon pump.
- Pulmonary Complications: Tracheal intubation or mechanical ventilation for more than 48 hours after surgery; need for tracheal reintubation associated with mechanical ventilation.
- Renal Complications: Serum creatinine level 2 mg/dL above the preoperative level; need for dialysis at any time postoperatively.
- Neurological Complications: Altered level of consciousness or coma occurring in association with neurological injury during surgery; sensory, motor, or reflex alterations at any time postoperatively.
- Infectious Complications: Considered pulmonary, urinary, surgical site infections, and bloodstream infections, according to the Diagnostic Criteria for Healthcare-Associated Infections by the Brazilian National Health Surveillance Agency.

Three risk categories were established: low (score of 0 to 5), moderate (score of 6 to 9), and high (score ≥ 10)^(6)^. An increase in the clinical risk score was associated with a higher frequency of individual complications.

### Data Analysis and Statistics

The data obtained from the medical records were entered and stored in an electronic spreadsheet using Microsoft Excel 2013, specifically created for this purpose, forming the study’s database. The construction of the database was thoroughly discussed among the authors, developed by the first author, and validated by a second researcher on the team and a statistician. Subsequently, the data were exported for statistical analysis to R for Windows version 6.4.2 (https://cran.r-project.org/bin/windows/base/).

Descriptive statistics were used to characterize the social and clinical profiles of the participants, with results presented as absolute numbers and simple percentages. Bivariate analysis was employed to examine the association between the risk score and complications using Fisher’s exact test. The confidence intervals for the relative risks between the Tuman Score and postoperative complications were obtained through Quasi-Poisson regression, with a 95% confidence level.

## RESULTS

Of the 130 cardiac surgical procedures performed during the period, 60 did not meet the inclusion criteria: 59 due to the type of procedure and one because the patient was under 18 years old. A total of 70 cardiac surgical procedures were selected and comprise the non-probabilistic final sample of this study.

According to the results presented in Table 1, the majority of patients were men (58.57%), aged between 41 and 64 years (50%). The most frequently performed cardiac surgical procedure was myocardial revascularization (50%), followed by valve replacement (42.86%) and myocardial revascularization associated with valve replacement (7.14%).

**Table 1:** Relationship between the Tuman Score and the social and clinical characteristics of patients undergoing cardiac surgical procedures at a University Hospital, Salvador, Bahia, Brazil, 2019-2020 (n=70)

Variables	n (%)	Tuman Score^*^			*p* value
		0-5 41 (58.57%)	6-9 27 (38.57%)	≥ 10 02 (2.85%)	
Age Range					
18 - 40	12 (17.14)	03	09	00	0.008
41 - 64	35 (50)	23	10	02	
65 - 74	16 (22.86)	13	03	00	
> 75	07 (10)	02	05	00	
Sex					
Male	41 (58.57)	30	11	00	0.003
Female	29 (41.43)	11	16	02	
Type of Surgery					
MR^**^	35 (50)	30	05	00	
Valve Replacement	30 (42.86)	09	20	01	0.000
MR^**^ and Valve Replacement	05 (7.14)	02	02	01	
Days of preoperative hospitalization					
< 10	32 (45.7)	19	13	00	0.578
11 - 20	18 (25.71)	12	05	01	
21 - 30	13 (18.6)	06	06	01	
> 31	07 (10)	04	03	00	
Days of postoperative ICU stay					
< 3	35 (50)	20	15	00	0.555
04 - 09	33 (47.14)	20	11	02	
> 10	02 (2.86)	01	01	00	
Days of perioperative period					
< 15	14 (20)	10	04	00	0.645
16 - 20	09 (12.86)	07	02	00	
21 - 30	20 (28.57)	11	08	01	
> 31	27 (38.57)	13	13	01	
Readmission to ICU					
Yes	07 (10)	04	03	00	1
No	63 (90)	37	24	02	
Outcome					
Hospital discharge	68 (97.14)	41	25	02	0.202
Death	02 (2.86)	00	02	00	

* Tuman Score: 0 to 5 low risk; 6 to 9 moderate risk; ≥ 10 high risk;

** Myocardial Revascularization.

More than half of the patients (67.14%, n=47) stayed in the hospital for more than 20 days during the entire perioperative period. Regarding the length of stay in the ICU postoperatively, it was observed that half of the patients stayed for less than three days (n=35), approximately 47.14% (n=33) stayed up to nine days, and 2.86% (n=2) stayed for more than ten days. Of these, 10% (n=7) required readmission to the ICU. The vast majority (97.14%, n=68) were discharged home.

Considering the Tuman Score (6) applied to evaluate the risk of postoperative complications, 58.57% (n=41) were classified as low risk, 38.57% (n=27) as moderate risk, and 2.85% (n=2) as high risk.

Analyzing the relationship between the Tuman Score and the patients’ social and clinical characteristics, an association was found between a lower risk of postoperative complications in patients aged 41 to 64 years (p=0.008), male (p=0.003), and those indicated for myocardial revascularization (p=0.000)

The relationship between postoperative complications in patients undergoing cardiac surgical procedures and the Tuman Score is presented in Table 2. The Relative Risk (RR) considered the moderate and high scores compared to the low score for complication risk, with a 95% confidence interval.

**Table 2:** Association between postoperative complications in patients undergoing cardiac surgical procedures and the Tuman Score at a University Hospital, Salvador, Bahia, Brazil, 2019-2020 (n=70)

Postoperative Complications	Tuman Score^*^	n	*p* value	Relative Risk (RR)	95% Confidence Interval
Cardiac Complications	0-5	14	-	1	-
	6-9	11	0.588	1.04	[0.90 - 1.20]
	≥ 10	02	0.625	1.65	[0.99 - 2.78]
Pulmonary Complications	0-5	01	-	1	-
	6-9	01	0.767	1.01	[0.96 -1.05]
	≥ 10	01	0.002	1.32	[1.12 -1.55]
Neurological Complications	0-5	03	-	1	-
	6-9	06	0.081	1.08	[0.99 - 1.18]
	≥ 10	01	0.047	1.20	[1.01 - 1.63]
Infectious Complications	0-5	08	-	1	-
	6-9	06	0.791	1.01	[0.91 - 1.13]
	≥ 10	01	0.314	1.20	[0.84 - 1.72]
Death	6-9	02	-	-	-

* *Tuman Score: 0 to 5 low risk; 6 to 9 moderate risk; ≥ 10 high risk; No renal complications or deaths were observed in the low and high-risk groups.*

Patients evaluated as high risk on the Tuman Score (≥ 10), compared to those with low risk (0-5), had a higher RR of cardiac (1.65) and pulmonary complications (1.32), followed by neurological (1.20) and infectious complications (1.20).

There was an association between high-risk patients and pulmonary complications (RR=1.32, p=0.002) and neurological complications (RR=1.20, p=0.047). Despite the RR of 1.65 for high-risk patients for cardiac complications, this association was not confirmed by bivariate analysis (p=0.625).

When observed individually, cardiac complications were the most frequent events (n=16), followed by infection (n=6). All postoperative pulmonary complications were simultaneous events with cardiac, neurological, and infectious complications.

Two patients died. Both were evaluated as moderate risk; one presented a cardiac complication, while the other presented all the investigated complications: cardiac, pulmonary, neurological, and infectious.

## DISCUSSION

Considering the sociodemographic and clinical characteristics of patients undergoing cardiac surgical procedures, there was a predominance of males aged between 41 and 64 years. In the external validation study of the Tuman Score in 2008, with 294 patients, 208 (70.3%) were male, aged between 20 and 84 years (mean ± standard deviation of 58.93 ± 11.79 years)^(10)^. Other similar studies have also found results that corroborate this finding^(11-16)^.

A study that investigated preoperative risk factors in 335 patients undergoing mechanical prosthesis implantation (valve surgery) identified that 54% of the patients were male; however, it did not find an association between sex and risk factors (p=0.262)^(12)^, unlike the results of this research (p=0.003).

The literature describes that being female is a risk factor for complications and mortality after cardiac surgical procedures due to a more advanced senility process, reduced estrogen with increasing age, and lower body mass^(11,16)^. However, men are considerably more subjected to these types of surgeries. Given this representation, it is important to reinforce the need to improve men’s knowledge, especially regarding chronic non-degenerative diseases.

Additionally, age is also a component that influences the risk factors for hospital morbidity. A study conducted from 2002 to 2007 sought to identify risk factors for postoperative morbidity in septuagenarian patients. The incidence of pulmonary, infectious, renal, and neurological complications was significantly higher in the group over 70 years old^(15)^. Retrospective research with patients between 1999 and 2012 showed that mortality was higher in women (17.3%; p<0.05) aged 70 or older (22.8%)^(16)^, corroborating the results of this study.

The higher incidence of postoperative complications and mortality in the elderly is a consequence of tissue fragility and limited functional reserve^(15)^. Additionally, the elderly population has a potential risk for coronary artery disease^(16-17)^. These conditions reaffirm that mortality increases significantly with age^(18)^. Therefore, while the recommendation of cardiac surgical procedures is debated in elderly patients, the recommendation for younger patients with early-diagnosed heart disease is considered a choice with a lower prevalence of risk factors^(13)^.

Furthermore, clinical factors also impact the outcomes, including the type of surgical procedure performed. In this sense, the predominance of myocardial revascularization in this study was also observed in others^(3,10-11,15,17)^.

The clinical conditions related to the most frequent comorbidities in preoperative patients were acute myocardial infarction less than three months prior, congestive heart failure, and pulmonary hypertension, identified by the Tuman Score. Another study conducted in São Paulo, Brazil, between 2013 and 2015, with 125 patients, showed that arterial hypertension, overweight/obesity, and dyslipidemia were the most predominant comorbidities^(14)^.

Risk factors for morbidity and mortality after cardiac surgical procedures are well-documented in the literature^(4,12-13,17,19)^. In this context, the Tuman Score was developed to evaluate and classify the risk of postoperative complications using preoperative severity factors.

Considering the risk classifications for complications after cardiac surgical procedures, the results presented here converge with the validation study of the Tuman Score conducted in São Paulo, where most patients (74.11%) were characterized as low risk; 21.57% as moderate risk; and 4.31% as high risk^(6)^. Other studies have also found similar results^(10,14)^.

According to Tuman^(6)^, individual complications, including operative mortality, also increase according to the risk score classification. Strabelli^(10)^ also demonstrated a statistically significant relationship between risk classification and a higher occurrence of complications (p=0.034). This study evidenced the association between high risk for postoperative complications and the occurrence of pulmonary (p=0.002) and neurological complications (p=0.047) in patients undergoing cardiac surgical procedures.

A meta-analysis found that postoperative complications tend to be higher in high-risk patients compared to those with lower risk^(20)^. High risk for postoperative complications may be related to individual predictive factors, such as advanced age, renal dysfunction, recent myocardial infarction, and pulmonary hypertension^(21)^.

A Danish study using the Tuman Score evaluated 628 patients and demonstrated that factors that can increase the risk of complications include previous valve surgery, advanced age, renal dysfunction, recent myocardial infarction, and pulmonary hypertension, and that the Score predicts good outcomes for low-risk patients^(21)^.

In the present research, the relative risk for postoperative complications increased in the moderate and high scores, and cardiac and infectious complications had a higher incidence when analyzed in isolation. Based on this, it is observed that the preoperative period is a crucial moment for patient involvement and risk assessment to avoid adverse reactions and possible postoperative complications^(22)^.

Tuman^(6)^ adds that scores closer to high risk are associated with longer ICU stays for patients. It was also found that patients with infectious complications had an association with longer ICU stays (p=0.001) and prolonged hospitalization (p=0.001)^(10)^.

In this study, the results did not demonstrate an association between the Tuman Score and prolonged ICU stay or ICU readmission. However, postoperative complications often require longer hospital stays^(17)^ and, at times, the need for ICU stay^(6,10,23-24)^. Due to the characteristics of ICU hospitalization, the risk of complications increases. Therefore, there is a need to reinforce unit routines, maintain multidisciplinary discussions for patient care, and define care strategies to reduce and control postoperative complications in patients undergoing cardiac surgical procedures.

Regarding ICU stay outcomes, it was observed that a large part (54.0%) of patients who stayed in the ICU for three days or more were discharged from the hospital (91.5%). Of those not readmitted to the ICU (96.3%), 11.3% died (p<0.002) and 88.7% were discharged from the hospital^(16)^. Systemic arterial hypertension, renal disease, and cardiovascular disease influenced outcomes and hospital readmission^(15-16)^.

Pulmonary and neurological complications were associated with the Tuman Score (p=0.002 and p=0.047, respectively), though the patients studied were predominantly cardiac. It is noteworthy that cardiac complications had a higher relative risk (1.65) for patients with a high-risk score.

Among the main cardiac clinical conditions, coronary insufficiency has a relative risk 1.5 times higher than in the group with valvular heart disease, and in the group with coronary insufficiency + valvular heart disease, a relative risk 1.8 times higher than in the group with valvular heart disease^(10)^. This demonstrates that cardiac events significantly influence cardiac complications, being much more prevalent (15.53%) than pulmonary (3.88%) and infectious complications (2.91%)^(17)^. Other studies also show that pulmonary complications are among the leading causes of early mortality after thoracic surgery (including cardiac surgery), significantly impacting patient outcomes^(25-27)^.

Regarding neurological complications, ischemic stroke is a frequent event after cardiac surgical procedures, mainly affecting older individuals and females. These patients present a higher risk of mortality, prolonged ICU stay, and hospital stay^(23)^.

Several risk assessment models for cardiac surgical procedures have been developed and validated to analyze mainly postoperative morbidity and mortality and hospital length of stay. This reveals a challenge and demonstrates that there is no universal and ideal system^(11)^. Risk assessment models have multifactorial indices and do not consider the critical intraoperative moment, which is the main challenge for health researchers^(5,10)^.

Regarding cardiac surgical procedures, other risk factors for postoperative complications were found, such as age ≥70 years, cardiogenic shock, ischemia, dialysis dependence, delay in accessing the healthcare system, and hospitalization^(11)^. Given this, it is noticeable that many other factors can impact risk scores for complications after cardiac surgical procedures and, consequently, the clinical outcomes of patients.

To facilitate the reduction of surgical risks and postoperative complications, healthcare services are encouraged to systematize patient care. Protocols are recommended to guide safe and high-quality care, ensure the necessary information, and develop strategies for managing problems and reducing adverse circumstances for patients^(28)^.

Nursing staff constitute the largest group in the healthcare team and provide continuous care to patients^(29)^. The nursing process methodologically structures patient care, including the identification and prevention of risks, while also considering global guidelines for patient safety during the perioperative period. In this way, the nursing process is essential to ensure safe and qualified care.

### Study limitations

This investigation has limitations due to the reduced sample size, resulting from the COVID-19 pandemic declared in March 2020. The hospital had to quickly adapt to accommodate infected patients and suspend elective procedures to increase bed availability and, above all, as a preventive measure to control the pandemic, as guided by regulatory bodies and adopted by healthcare services and institutions^(30-31)^.

### Contributions to Nursing

It is believed that the presented results can support further studies on risk assessment for postoperative complications in cardiac surgical procedures. Additionally, they can foster the development and incorporation of risk scores in clinical practice, aiming at the early identification of factors complicating these patients’ health and guiding and improving the quality of care provided by healthcare professionals, especially cardiovascular nurses.

Nurses working in cardiology must be able to adequately assess the general and specific clinical conditions of patients. Therefore, the knowledge presented in this study contributes to enabling these professionals to assist decisively in care management, enhancing the quality of information and making more assertive and dynamic decisions according to the individual needs of patients.

## CONCLUSIONS

The study evaluated the risks of postoperative complications in patients undergoing cardiac surgical procedures using the Tuman Score and found that most patients, especially men, aged between 41 and 64 years and in the preoperative period of myocardial revascularization, had a low risk for developing complications. Pulmonary and neurological complications were associated with patients evaluated with a high-risk score. Cardiac complications, followed by infectious ones, were the most prevalent among the patients. The outcome was favorable for hospital discharge.

Patient safety in the hospital environment is essential to provide quality healthcare and reduce morbidity and mortality. The risk score for postoperative complications guides nurses and other healthcare team members in providing individualized care that considers the risks early in the preoperative period.
