# Supplementary material for: Risk assessment for postoperative complications in patients undergoing cardiac surgical procedures
Source: Rev Bras Enferm. 2024 Sep 20;77(4):e20230127. doi: 10.1590/0034-7167-2023-0127 (PMC11419680; doi:10.1590/0034-7167-2023-0127)
Supplement: Translation

# Translation: Avaliação de risco para complicações pós-operatórias em pacientes submetidos a procedimentos cirúrgicos cardíacos

**DOI:** 10.1590/0034-7167-2023-0127pt

**Authors:** Carolina Larrosa de Almeida, Jones Sidnei Barbosa de Oliveira, Cláudia Geovana da Silva Pires, Cláudia Silva Marinho

**Affiliations:** I Universidade Federal da Bahia. Salvador, Bahia, Brasil

**Keywords:** Doenças Cardiovasculares, Procedimentos Cirúrgicos Cardíacos, Complicações Pós-Operatórias, Avaliação de Risco, Enfermagem Cardiovascular.

## Abstract

**Objetivos:**

avaliar o risco de complicações pós-operatórias em pacientes cardíacos.

**Métodos:**

pesquisa avaliativa, utilizando o Escore de Tuman, em prontuários de 70 pacientes adultos submetidos a cirurgia cardíaca em um Hospital Universitário. Foi utilizado o software R for Windows para as análises. A estatística descritiva e a análise bivariada foram utilizadas para verificar a associação entre escore de risco e complicações. O risco relativo entre o Escore de Tuman e as complicações pós-operatórias foi obtido por meio de regressão Quasi-Poisson, com intervalo de confiança de 95%.

**Resultados:**

predominou paciente do sexo masculino (58,57%), com idades entre 41-64 anos (50%), que realizaram Revascularização do Miocárdio (50%), e esses foram associados ao menor risco de complicações pós-operatórias (p=0,003), (p=0,008) e (p=0,000), respectivamente. Os pacientes de alto risco tiveram complicações pulmonares (RR=1,32 e p=0,002) e complicações neurológicas (RR=1,20 e p=0,047).

**Conclusões:**

a avaliação dos riscos pré-operatórios promove uma assistência qualificada para reduzir as complicações pós-operatórias.

## INTRODUÇÃO

No pré-operatório de procedimentos cirúrgicos cardíacos, é importante estimar a probabilidade de desfechos adversos. No Brasil, foram realizadas cerca de 24.000 cirurgias cardíacas em 2022. No mesmo ano, 642 pacientes foram internados para tratamento de complicações após esses procedimentos. Quando comparado ao ano anterior, observa-se uma tendência ascendente na realização desse tipo de cirurgia, assim como no aumento das complicações pós-operatórias, com 19.632 procedimentos cirúrgicos cardíacos realizados e 329 complicações^(1)^. As complicações mais comuns foram a redução do débito urinário, arritmias cardíacas e hipertensão^(2)^.

Nesse contexto, o uso dos escores de risco instrumentaliza os profissionais da saúde a estimarem o prognóstico para complicações dos pacientes. As complicações advindas da assistência à saúde geram grande impacto na saúde individual dos pacientes, além do impacto econômico para o sistema de saúde.

Desse modo, é essencial a utilização de estratégias de identificação de pacientes com maior risco de complicações e a adoção de medidas para preveni-las ou minimizá-las. Estudos publicados descrevem alguns escores e índices de risco utilizados em todo o mundo^(3-5)^. Este estudo utilizou o Escore de Tuman^(6)^ por ser um instrumento amplamente utilizado e validado por pesquisadores brasileiros para avaliar o risco e identificar seus fatores para complicações pós-operatórias em pacientes submetidos a procedimentos cirúrgicos cardíacos, com um método prático composto de variáveis de fácil obtenção.

Esse estudo investigativo é importante, uma vez que possibilita identificar o perfil clínico dos pacientes submetidos a procedimentos cirúrgicos cardíacos, as complicações que ocorrem no pós-operatório, além de estimar o risco para a ocorrência dessas complicações. Isso instrumentaliza a equipe de enfermagem e demais profissionais da equipe de saúde com conhecimento para o desenvolvimento de ações visando qualificar suas práticas, fluxos de atendimento, protocolos de cuidado e planos de cuidados individuais para esses pacientes.

Além disso, foi evidenciada uma lacuna na literatura, onde a grande maioria dos estudos identifica e descreve repetidamente os riscos pós-operatórios como situações adversas, mas poucos evidenciam condutas precoces para prevenção e controle dos riscos pós-operatórios.

Nesse contexto, este estudo tem um caráter inovador e original, pois aplica, discute e divulga uma tecnologia de cuidado, um instrumento sistematizado de avaliação precoce de riscos para complicações pós-operatórias em pacientes cardíacos, validado no Brasil e de uso simplificado para a prática da enfermagem e demais profissionais de saúde.

## OBJETIVOS

Avaliar o risco de complicações pós-operatórias em pacientes submetidos a procedimentos cirúrgicos cardíacos por meio do Escore de Tuman.

## MÉTODOS

### Aspectos Éticos

Esta pesquisa atendeu às diretrizes das Resoluções 466/2012 e 510/2016 do Conselho Nacional de Saúde sobre pesquisa envolvendo seres humanos^(7-8)^ e às orientações da Carta Circular nº. 039/2011/CONEP/CNS/GB/MS, que trata do Uso de Dados de Prontuários para Fins de Pesquisa no Brasil^(9)^. O consentimento do paciente foi dispensado por se tratar de uma pesquisa retrospectiva de base documental. Obteve aprovação pelo Comitê de Ética em Pesquisa (CEP) do Hospital Universitário Professor Edgar Santos (HUPES).

### Desenho, Período e Local do Estudo

Pesquisa avaliativa, de natureza observacional e retrospectiva, que investigou todos os pacientes submetidos a procedimentos cirúrgicos cardíacos em um hospital universitário, na cidade de Salvador, Bahia, no período de 1 de janeiro de 2019 a 31 de dezembro de 2020. O Hospital Universitário, de grande porte, é referência no estado para o atendimento de pacientes cardíacos e conta com serviços especializados como Ambulatórios, Enfermaria de Cardiologia, Unidade de Terapia Intensiva Cardiológica, Hemodinâmica e Centro Cirúrgico.

A coleta dos dados foi realizada nos prontuários, eletrônicos e físicos, dos pacientes, no período de 1 de fevereiro a 31 de março de 2021, por uma das autoras, enfermeira residente, que atuava na Unidade de Clínica Cirúrgica Cardiológica. Este manuscrito é produto de seu Trabalho de Conclusão de Curso. Para a elaboração do manuscrito, foram seguidas as recomendações do *Strengthening the Reporting of Observational Studies in Epidemiology* (STROBE).

### Amostra e Critérios de Elegibilidade

No período de interesse, foram realizados 130 procedimentos cirúrgicos cardíacos, dos quais 70 foram selecionados para o estudo. Os critérios de inclusão considerados foram: cirurgias em pacientes com idade igual ou superior a 18 anos, de ambos os sexos, submetidos a: cirurgia valvar (troca ou plástica); cirurgia de revascularização coronariana (com ou sem a utilização de circulação extracorpórea); e cirurgia associada (cirurgia de revascularização coronariana + cirurgia valvar). Informações de pacientes fora desses critérios de elegibilidade definidos foram excluídas da pesquisa.

### Protocolo do estudo

A coleta de dados foi realizada por meio de registros nos prontuários dos pacientes. Os dados de interesse referem-se às características da internação: período de internação (tempo de pré-operatório e tempo de pós-operatório em Unidade de Terapia Intensiva - UTI - e em Enfermaria); e às variáveis (sociais e clínicas) para o cálculo do Escore de Risco de Tuman: caráter de urgência da cirurgia; idade; sexo; condições prévias de saúde (disfunção renal; infarto agudo do miocárdio há 3-6 meses, há menos de 3 meses ou se não houve; cirurgia cardíaca; hipertensão pulmonar; antecedentes de doença cerebrovascular; insuficiência cardíaca congestiva; disfunção do ventrículo esquerdo); tipo de procedimento cirúrgico cardíaco a ser realizado: troca valvar múltipla ou revascularização do miocárdio associada a troca valvar; e indicação da válvula cardíaca a ser reparada: aórtica ou mitral^(6)^.

O Escore de Tuman é um modelo de predição de risco de complicação no pós-operatório de procedimentos cirúrgicos cardíacos, desenvolvido no Departamento de Anestesiologia, Cardiologia e Cirurgia Torácica em Chicago, Estados Unidos, em 1992. O objetivo foi criar um modelo para estratificar o risco de morbidade após procedimentos cirúrgicos cardíacos em adultos, por meio de dados clínicos objetivos e prontamente disponíveis^(6)^.

Dessa forma, foram consideradas suas definições sobre as categorias a serem avaliadas: infarto agudo do miocárdio, hipertensão pulmonar, doença cerebrovascular, insuficiência cardíaca congestiva, disfunção do ventrículo esquerdo e disfunção renal, assim como as complicações cardíacas, pulmonares, renais, neurológicas e infecciosas^(6)^.

- Infarto agudo do miocárdio: Paciente apresentando dois ou mais dos seguintes achados: elevação da creatinofosfoquinase-fração de membrana (CKMB); ondas Q novas ao eletrocardiograma; falhas na captação do pirofosfato de tecnécio na cintilografia do miocárdio.
- Hipertensão pulmonar: Pressão sistólica da artéria pulmonar ≥30 mmHg evidenciada ao ecocardiograma ou durante estudo hemodinâmico.
- Doença cerebrovascular: Antecedente de acidente vascular cerebral e/ou alterações vasculares diagnosticadas durante carotidoangiografia.
- Insuficiência cardíaca congestiva: Alterações radiológicas compatíveis com congestão pulmonar ou presença de terceira bulha cardíaca.
- Disfunção do ventrículo esquerdo: Fração de ejeção menor que 35% ao ecocardiograma.
- Disfunção renal: Creatinina sérica maior que 1,4 mg/dl (miligramas por decilitro).
- Complicações cardíacas:
- Infarto agudo do miocárdio perioperatório: Paciente apresentando dois ou mais dos seguintes: elevação da CKMB; ondas Q novas ao eletrocardiograma; falhas na captação do pirofosfato de tecnécio na cintilografia do miocárdio.
- Síndrome de baixo débito cardíaco: Índice cardíaco menor que dois litros por minuto por metro quadrado, com necessidade de drogas inotrópicas por mais de duas horas e/ou uso de balão intra-aórtico.
- Complicações pulmonares: Intubação traqueal ou ventilação mecânica por mais de 48 horas após a cirurgia; necessidade de reintubação traqueal associada à ventilação mecânica.
- Complicações renais: Nível sérico de creatinina de 2 mg/dl acima do nível pré-operatório; necessidade de diálise em qualquer momento do pós-operatório.
- Complicações neurológicas: Alteração do nível de consciência ou coma ocorrendo em associação com lesão neurológica durante a cirurgia; alteração sensorial, motora ou de reflexos em qualquer momento do pós-operatório.
- Complicações infecciosas: Foram consideradas as infecções pulmonares, urinárias, da ferida operatória e as infecções da corrente sanguínea, de acordo com os Critérios Diagnósticos de Infecção Relacionada à Assistência à Saúde da Agência Nacional de Vigilância Sanitária do Brasil.

Foram estabelecidas 3 categorias de risco: baixo (escore de 0 a 5), moderado (escore de 6 a 9); e alto (escore ≥ 10)^(6)^. O aumento do escore de risco clínico foi associado a uma maior frequência de complicações individuais.

### Análise dos resultados e estatística

Os dados obtidos nos prontuários foram digitados e armazenados em planilha eletrônica no *Software Microsoft Excel*, versão 2013, criada para este fim e compondo o banco de dados do estudo. A construção do banco de dados foi discutida amplamente entre os autores, desenvolvido pela primeira autora e validado por uma segunda pesquisadora da equipe e uma estatística. Após, para processamento das análises estatísticas foi exportado para o *Software R for Windows* versão 6.4.2 (https://cran.r-project.org/bin/windows/base/).

A estatística descritiva foi utilizada para caracterização social e clínica dos participantes e seus resultados apresentados em números absolutos e percentuais simples. A análise bivariada foi utilizada para analisar a associação entre o escore de risco e complicações por meio do teste Exato de Fisher. Os intervalos de confiança dos riscos relativos entre Escore de Tuman e as Complicações pós-operatórias foram obtidos pela regressão de Quase-Poisson, considerando um nível de confiança de 95%.

## RESULTADOS

Dos 130 procedimentos cirúrgicos cardíacos realizados no período, 60 não atenderam aos critérios de inclusão, sendo 59 devido ao tipo de procedimento e um porque o paciente era menor de 18 anos. Um total de 70 procedimentos cirúrgicos cardíacos foram selecionados e compõem a amostra final não probabilística deste estudo.

De acordo com os resultados apresentados na Tabela 1, a maioria dos pacientes eram homens (58,57%), na faixa etária entre 41 e 64 anos (50%). O procedimento cirúrgico cardíaco realizado com maior frequência foi a revascularização do miocárdio (50%), seguida pela troca de válvula (42,86%) e pela revascularização do miocárdio associada à troca de válvula (7,14%).

**Tabela 1:** Relação entre Escore de Tuman e as características sociais e clínicas de pacientes submetidos a procedimentos cirúrgicos cardíacos em um Hospital Universitário, Salvador, Bahia, Brasil, 2019-2020 (n=70)

Variáveis	n (%)	Escore de Tuman^*^			Valor de *p*
		0-5 41 (58,57%)	6-9 27 (38,57%)	≥ 10 02 (2,85%)	
Faixa Etária					
18 - 40	12 (17,14)	03	09	00	0,008
41 - 64	35 (50)	23	10	02	
65 - 74	16 (22,86)	13	03	00	
> 75	07 (10)	02	05	00	
Sexo					
Masculino	41 (58,57)	30	11	00	0,003
Feminino	29 (41,43)	11	16	02	
Tipo de Cirurgia					
RM^**^	35 (50)	30	05	00	
Troca Valvar	30 (42,86)	09	20	01	0,000
RM^**^ e Troca Valvar	05 (7,14)	02	02	01	
Dias de internação pré-operatório					
< 10	32 (45,7)	19	13	00	0,578
11 - 20	18 (25,71)	12	05	01	
21 - 30	13 (18,6)	06	06	01	
> 31	07 (10)	04	03	00	
Dias de internação do pós-operatório em UTI					
< 3	35 (50)	20	15	00	0,555
04 - 09	33 (47,14)	20	11	02	
> 10	02 (2,86)	01	01	00	
Dias do perioperatório					
< 15	14 (20)	10	04	00	0,645
16 - 20	09 (12,86)	07	02	00	
21 - 30	20 (28,57)	11	08	01	
> 31	27 (38,57)	13	13	01	
Reinternação em UTI					
Sim	07 (10)	04	03	00	1
Não	63 (90)	37	24	02	
Desfecho					
Alta hospitalar	68 (97,14)	41	25	02	0,202
Óbito	02 (2,86)	00	02	00	

* Escore de Tuman: de 0 a 5 baixo risco; 6 a 9 risco moderado; ≥ 10 alto risco;

** Revascularização do Miocárdio.

Mais da metade dos pacientes (67,14%, n=47) permaneceram no hospital por mais de 20 dias considerando todo o período perioperatório. Em relação ao tempo de internação dos pacientes no pós-operatório em UTI, observou-se que metade dos pacientes ficou menos de três dias (n=35), cerca de 47,14% (n=33) ficaram até nove dias e 2,86% (n=2) permaneceram por mais de dez dias. Destes, 10% (n=7) necessitaram de reinternação na UTI. A grande maioria (97,14%, n=68) recebeu alta hospitalar para casa.

Ao considerar o Escore de Tuman (6) aplicado para avaliação do risco de complicações pós-operatórias, 58,57% (n=41) foram classificados como de baixo risco, 38,57% (n=27) com risco moderado e 2,85% (n=2) com alto risco.

Ao analisar a relação entre o Escore de Tuman e as características sociais e clínicas dos pacientes, foi encontrada associação entre o menor risco de complicações pós-operatórias em pacientes na faixa etária de 41 a 64 anos (p=0,008), do sexo masculino (p=0,003) e com indicação para revascularização do miocárdio (p=0,000).

A relação entre as complicações pós-operatórias de pacientes submetidos a procedimentos cirúrgicos cardíacos e o Escore de Tuman é apresentada na Tabela 2. O Risco Relativo (RR) considerou os escores moderado e alto em comparação com o escore baixo para risco de complicações, considerando um intervalo de confiança de 95%.

**Tabela 2:** Associação entre as complicações pós-operatórias de pacientes submetidos a procedimentos cirúrgicos cardíacos e o Escore de Tuman, em um Hospital Universitário, Salvador, Bahia, Brasil, 2019-2020 (n=70)

Complicações pós-operatórias	Escore de Tuman^*^	n	Valor de *p*	Risco Relativo (RR)	Intervalo de Confiança 95%
Complicações Cardíacas	0-5	14	-	1	-
	6-9	11	0,588	1,04	[0,90 - 1,20]
	≥ 10	02	0,625	1,65	[0,99 - 2,78]
Complicações Pulmonares	0-5	01	-	1	-
	6-9	01	0,767	1,01	[0,96 -1,05]
	≥ 10	01	0,002	1,32	[1,12 -1,55]
Complicações Neurológicas	0-5	03	-	1	-
	6-9	06	0,081	1,08	[0,99 - 1,18]
	≥ 10	01	0,047	1,20	[1,01 - 1,63]
Complicações Infecciosas	0-5	08	-	1	-
	6-9	06	0,791	1,01	[0,91 - 1,13]
	≥ 10	01	0,314	1,20	[0,84 - 1,72]
Óbito	6-9	02	-	-	-

* *Escore de risco: de 0 a 5 baixo risco; 6 a 9 risco moderado; ≥ 10 alto risco; Não houve Complicações Renais e não houve óbitos no baixo e alto risco.*

Os pacientes avaliados como de alto risco no Escore de Tuman (≥ 10), comparados aos de baixo risco (0-5), apresentaram maior RR de complicações cardíacas (1,65) e pulmonares (1,32), seguidas de complicações neurológicas (1,20) e infecciosas (1,20).

Foi evidenciada a associação entre pacientes de alto risco e complicações pulmonares (RR=1,32 e p=0,002) e complicações neurológicas (RR=1,20 e p=0,047). Apesar do RR de 1,65 dos pacientes de alto risco para complicações cardíacas, essa associação não foi comprovada pela análise bivariada (p=0,625).

Quando observadas isoladamente, a complicação cardíaca foi o evento mais frequente (n=16), seguido por infecção (n=6). Todas as complicações pulmonares pós-operatórias foram eventos simultâneos às complicações cardíacas, neurológicas e infecciosas.

Dois pacientes evoluíram para óbito. Ambos foram avaliados como de risco moderado; um deles apresentou uma complicação cardíaca, enquanto o outro apresentou todas as complicações investigadas: cardíaca, pulmonar, neurológica e infecciosa.

## DISCUSSÃO

Considerando as características sociodemográficas e clínicas dos pacientes submetidos a procedimentos cirúrgicos cardíacos, notou-se a predominância do sexo masculino, na faixa etária de 41 a 64 anos. No estudo de validação externa do Escore de Tuman em 2008, com 294 pacientes, 208 (70,3%) eram do sexo masculino, com idades entre 20 e 84 anos (média ± desvio-padrão de 58,93 ± 11,79 anos)^(10)^. Outros estudos semelhantes também trouxeram resultados que corroboram esse achado^(11-16)^.

Um estudo que investigou fatores de risco pré-operatórios em 335 pacientes submetidos a implante de prótese mecânica (cirurgia valvar) identificou que 54% dos pacientes eram do sexo masculino, no entanto, não encontrou associação entre o sexo e os fatores de risco (p=0,262)^(12)^, diferentemente dos resultados desta pesquisa (p=0,003).

A literatura descreve que o sexo feminino é um fator de risco para complicação e mortalidade após procedimentos cirúrgicos cardíacos, devido ao processo de senilidade mais avançado, redução do estrógeno com o aumento da idade e menor massa corporal^(11,16)^, mas os homens são consideravelmente mais submetidos a esses tipos de cirurgias. Diante dessa representatividade, é importante reforçar a necessidade de melhorar o conhecimento dos homens, especialmente no tocante às doenças crônicas não degenerativas.

Além disso, a idade também é um componente que influencia os fatores de risco para morbidade hospitalar. Um estudo, realizado de 2002 a 2007, buscou conhecer fatores de risco para morbidade pós-operatória em pacientes septuagenários. A incidência de complicações pulmonares, infecciosas, renais e neurológicas foi significativamente maior no grupo com mais de 70 anos^(15)^. Pesquisa retrospectiva, com pacientes entre 1999 e 2012, evidenciou que a mortalidade foi maior em mulheres (17,3%; p<0,05), com idade igual ou superior a 70 anos (22,8%)^(16)^, corroborando os resultados deste estudo.

A maior incidência de complicações e mortalidade pós-operatória em idosos é consequência da fragilidade tecidual e reserva funcional limitada^(15)^. Além disso, os idosos constituem uma população com risco potencial para doença arterial coronariana^(16-17)^. Essas condições reafirmam que a mortalidade aumenta significativamente com a idade^(18)^. Portanto, enquanto nos pacientes idosos a recomendação dos procedimentos cirúrgicos cardíacos é discutida, a recomendação para pacientes mais jovens e com doença cardíaca diagnosticada precocemente é considerada uma escolha com menor prevalência de fatores de risco^(13)^.

Além desses, os fatores clínicos também impactam, dentre eles o tipo de procedimento cirúrgico realizado. Nesse sentido, a predominância de revascularização do miocárdio neste estudo também foi observada em outros^(3,10-11,15,17)^.

As condições clínicas relacionadas às comorbidades mais frequentes nos pacientes no pré-operatório foram infarto agudo do miocárdio prévio a menos de três meses, insuficiência cardíaca congestiva e hipertensão pulmonar, identificadas pelo Escore de Tuman. Outro estudo, realizado em São Paulo, Brasil, entre 2013 e 2015, com 125 pacientes, demonstrou que hipertensão arterial, sobrepeso/obesidade e dislipidemia foram as comorbidades mais predominantes^(14)^.

Os fatores de risco para morbimortalidade após procedimentos cirúrgicos cardíacos são amplamente documentados na literatura^(4,12-13,17,19)^. Nesse sentido, o Escore de Tuman foi desenvolvido com o objetivo de avaliar e classificar em níveis o risco de complicações pós-operatórias, utilizando fatores de gravidade pré-operatórios.

Ao se considerar as classificações de risco para complicações após procedimentos cirúrgicos cardíacos, os resultados apresentados aqui convergem com o estudo de validação do Escore de Tuman, realizado em São Paulo, onde a maioria dos pacientes (74,11%) foi caracterizada como de baixo risco; 21,57% como de risco moderado; e 4,31% como de alto risco^(6)^. Outros estudos também encontraram resultados semelhantes^(10,14)^.

Para Tuman^(6)^, as complicações individuais, incluindo a mortalidade operatória, também aumentam de acordo com a classificação do Escore de Risco. Strabelli^(10)^ também evidenciou, em seu estudo, uma relação estatisticamente significativa entre a classificação dos riscos e a maior ocorrência de complicações (p=0,034). Neste estudo, foi evidenciada a associação entre o alto risco para complicações pós-operatórias e a ocorrência de complicações pulmonares (p=0,002) e neurológicas (p=0,047) em pacientes submetidos a procedimentos cirúrgicos cardíacos.

Uma metanálise verificou que as complicações pós-operatórias tendem a ser maiores em pacientes de alto risco quando comparados a riscos menores^(20)^. O alto risco para complicações pós-operatórias pode estar relacionado a fatores preditores do indivíduo, como idade avançada, disfunção renal, infarto do miocárdio recente e hipertensão pulmonar^(21)^.

Um estudo dinamarquês, utilizando o Escore de Tuman, avaliou 628 pacientes e demonstrou que os fatores que podem aumentar o risco de complicações incluem: cirurgia valvar prévia, idade avançada, disfunção renal, infarto do miocárdio recente e hipertensão pulmonar, e que o Escore prevê bons resultados para pacientes com baixo risco^(21)^.

Na presente pesquisa, o risco relativo para complicações pós-operatórias aumentou nos escores moderado e alto, e as complicações cardíacas e infecciosas apresentaram maior incidência quando analisadas de forma isolada. Com base nisso, observa-se que o período pré-operatório é um momento crucial para o envolvimento do paciente e a avaliação de riscos, a fim de evitar reações adversas e possíveis complicações no pós-operatório^(22)^.

Tuman^(6)^ acrescenta que a pontuação mais próxima ao risco alto é associada à maior duração de permanência dos pacientes na UTI. Verificou-se também que os pacientes com complicações infecciosas tiveram associação com o maior tempo de permanência na UTI (p=0,001) e internação mais prolongada (p=0,001^)(10)^.

Neste estudo, os resultados não demonstraram associação entre o Escore de Tuman e o tempo prolongado em UTI e a reinternação na UTI. No entanto, as complicações pós-operatórias demandam um maior tempo de internação^(17)^ e, por vezes, a necessidade de permanência em UTI^(6,10,23-24)^. A internação do paciente em UTI, devido às suas características, aumenta o risco de complicações. Dessa forma, há necessidade de reforçar as orientações sobre a rotina da unidade, manter as discussões multiprofissionais para o cuidado a cada paciente, bem como definir estratégias assistenciais para redução e controle das complicações pós-operatórias em pacientes submetidos a procedimentos cirúrgicos cardíacos.

Considerando o desfecho da permanência em UTI, foi observado que grande parte (54,0%) dos pacientes que permaneceram na UTI por três dias ou mais evoluiu para alta hospitalar (91,5%). Daqueles que não foram reinternados na UTI (96,3%), 11,3% evoluíram a óbito (p<0,002) e 88,7% tiveram alta hospitalar^(16)^. A hipertensão arterial sistêmica, doença renal e doença cardiovascular influenciaram no desfecho e na readmissão hospitalar^(15-16)^.

As complicações pulmonares e neurológicas tiveram associação com o Escore de Tuman (p=0,002 e p=0,047, respectivamente), contudo, os pacientes estudados foram majoritariamente cardiológicos. Ressalta-se que as complicações cardíacas obtiveram maior risco relativo (1,65) para pacientes com Escore de risco elevado.

Dentre as principais condições clínicas cardíacas, a insuficiência coronariana possui um risco relativo 1,5 vezes maior que no grupo com cardiopatia valvar, e no grupo com insuficiência coronariana + cardiopatia valvar, um risco relativo 1,8 vezes maior que no grupo com cardiopatia valvar^(10)^. Isso demonstra que os eventos cardíacos influenciam sobremaneira nas complicações cardíacas, sendo muito mais prevalentes (15,53%) do que as pulmonares (3,88%) e infecciosas (2,91%)^(17)^. Outros estudos também demonstram que as complicações pulmonares estão entre as principais causas de mortalidade precoce após cirurgia torácica (incluindo as cardíacas), associando-as a um impacto significativo no desfecho dos pacientes^(25-27)^.

Em relação às complicações neurológicas, o acidente vascular isquêmico é um evento frequente após procedimentos cirúrgicos cardíacos, acometendo principalmente indivíduos de idade mais avançada e do sexo feminino. Esses pacientes apresentam maior risco de mortalidade, tempo prolongado de permanência em UTI e internação hospitalar^(23)^.

Alguns modelos de avaliação de risco para procedimentos cirúrgicos cardíacos foram desenvolvidos e validados para analisar principalmente a morbimortalidade pós-operatória e o tempo de internação hospitalar. Isso revela um desafio e demonstra que não há um sistema de uso universal e ideal^(11)^. Os modelos de avaliação de risco possuem índices multifatoriais e não consideram o momento decisivo do intraoperatório, sendo esse o principal desafio para os pesquisadores da área de saúde^(5,10)^.

Em relação aos procedimentos cirúrgicos cardíacos, foram encontrados outros fatores de risco para as complicações pós-operatórias, tais como: idade ≥70 anos, choque cardiogênico, isquemia, dependência de diálise, demora no acesso ao sistema de saúde e internação^(11)^. Diante disso, é perceptível que muitos outros fatores podem impactar os escores de risco para complicações após procedimentos cirúrgicos cardíacos e, consequentemente, no desfecho da condição clínica dos pacientes.

Como mecanismo para viabilizar a redução dos riscos cirúrgicos e das complicações pós-operatórias, os serviços de saúde são estimulados a sistematizar a assistência à saúde. São recomendados protocolos para orientar uma assistência segura e de qualidade, garantir as informações necessárias, além de desenvolver estratégias no manejo de problemas e reduzir circunstâncias adversas para o paciente^(28)^.

As trabalhadoras em enfermagem compõem a equipe de saúde em maior número e prestam assistência em tempo integral aos pacientes^(29)^. O processo de enfermagem estrutura metodologicamente os cuidados aos pacientes, entre eles aqueles relacionados à identificação e prevenção de riscos, considerando também as diretrizes mundiais para segurança do paciente durante o período perioperatório. Dessa forma, o processo de enfermagem é essencial para garantir uma assistência segura e qualificada.

### Limitações do estudo

Esta investigação apresenta limitações devido ao reduzido tamanho da amostra, em virtude da pandemia de COVID-19, decretada em março de 2020, que levou a instituição hospitalar a se readequar rapidamente para receber os pacientes infectados e a suspender os procedimentos eletivos como medida de aumentar a disponibilidade de leitos e, sobretudo, como medida preventiva de controle da pandemia, orientada pelos órgãos reguladores e adotada pelos serviços e instituições de saúde^(30-31)^.

### Contribuições para a área da enfermagem

Acredita-se que os resultados apresentados possam subsidiar a realização de outros estudos sobre a avaliação de risco para complicações pós-operatórias em procedimentos cirúrgicos cardíacos, além de fomentar o desenvolvimento e a incorporação dos escores de risco na prática assistencial, visando à identificação precoce de fatores complicadores da saúde desses pacientes, assim como orientar e qualificar a assistência ofertada pelos profissionais da saúde, sobretudo pelas trabalhadoras em enfermagem cardiovascular.

O profissional enfermeiro que atua na cardiologia deve ser capaz de avaliar adequadamente a condição clínica, geral e específica, dos pacientes. Portanto, o conhecimento apresentado no estudo contribui para que esse profissional auxilie de maneira decisiva na gestão do cuidado, favorecendo a qualidade das informações e, com isso, tomadas de decisões mais assertivas e dinâmicas de acordo com as necessidades individuais dos pacientes.

## CONCLUSÕES

O estudo avaliou os riscos de complicações pós-operatórias em pacientes submetidos a procedimentos cirúrgicos cardíacos por meio do Escore de Tuman e evidenciou que a maioria dos pacientes, especialmente homens, entre 41 e 64 anos e em pré-operatório de revascularização do miocárdio, apresentou baixo risco para o desenvolvimento de complicações. As complicações pulmonares e neurológicas estiveram associadas aos pacientes avaliados com escore de alto risco. As complicações cardíacas, seguidas das infecciosas, foram as mais presentes nos pacientes. O desfecho foi favorável para alta hospitalar.

A segurança do paciente no ambiente hospitalar é essencial para oferecer uma assistência em saúde de qualidade e reduzir a morbimortalidade. O escore de risco para complicações pós-operatórias orienta as enfermeiras e demais profissionais da equipe de saúde na prestação de uma assistência singular, que considera precocemente, no pré-operatório, os riscos após o procedimento cirúrgico.
